# Draft Genome Sequence of *Raoultella ornithinolytica* TNT, a Trinitrotoluene-Denitrating and Plant Growth-Promoting Strain Isolated from Explosive-Contaminated Soil

**DOI:** 10.1128/genomeA.00491-14

**Published:** 2014-05-29

**Authors:** Sofie Thijs, Jonathan Van Hamme, Panagiotis Gkorezis, Francois Rineau, Nele Weyens, Jaco Vangronsveld

**Affiliations:** a Hasselt University, Centre for Environmental Sciences, Agoralaan Building D, Diepenbeek, Belgium; b Thompson Rivers University, Department of Biological Sciences, Kamloops, British Columbia, Canada

## Abstract

We report the draft genome of *Raoultella ornithinolytica* TNT, a Gram-negative bacterium of the *Enterobacteriaceae* isolated from military soil in Belgium. Strain TNT uses nitrite released from trinitrotoluene (TNT) for growth and is a potent plant growth promoter. An analysis of its 5.6-Mb draft genome will bring insights into TNT degradation-reinforcing bioremediation applications.

## GENOME ANNOUNCEMENT

Trinitrotoluene (TNT)-contaminated sites pose risks to human and environmental health, making remediation a priority. Detoxification by indigenous bacteria or use of TNT-transforming bacteria in bioaugmented rhizoremediation is preferred to invasive and costly excavation or incineration techniques.

A TNT-detoxifying strain was isolated from TNT-contaminated forest soil at a military site in Belgium. Identified as *Raoultella ornithinolytica* B6 by partial 16S rRNA gene sequencing and phenotypic profiling, the closest related partial 16S rRNA gene sequence (97.8%) was from strain B6 (GenBank accession no. CP004142) (1).

Here, genomic DNA was isolated using a DNeasy blood and tissue kit (Qiagen, Venlo, the Netherlands), treated with RNase I, and purified by phenol:chloroform extraction. Purified DNA was digested and sequencing adaptors ligated using an Ion Xpress Plus fragment library kit (Life Technologies Inc., Burlington, Ontario, Canada). Adaptor-ligated DNA was size selected (480 bp) on a 2% E-Gel SizeSelect agarose gel, and Agencourt AMPure XP beads (Beckman Coulter, Mississauga, Ontario, Canada) were used for purifications. An Ion library quantitation kit was used prior to amplification and enrichment with an Ion PGM Template OT2 400 kit on an Ion OneTouch 2 system. An Ion Sphere quality control kit was used prior to sequencing with an Ion PGM 400 sequencing kit and a single 316v2 Chip on an Ion Torrent PGM (Life Technologies, Inc., Carlsbad, CA).

In total, 2.49 million reads (mean length, 282 bases) generated 703 Mb of data, of which 938,910 reads were assembled using MIRA version 3.9.9 (2) into 39 contigs, giving a consensus length of 5,652,765 bp at 48× coverage. Mauve (3) was used to order the contigs and compare the genome with the closest related reference genome, that of *R. ornithinolytica* B6 (accession no. CP004142). The annotation was completed using the PGAP (NCBI) pipeline (4). The genome of *R. ornithinolytica* TNT consists of a single circular chromosome (55.5% G+C content), which includes 4,935 coding genes, 180 pseudogenes, 34 rRNAs (5S, 16S, 23S), 80 tRNAs, and 7 noncoding RNAs (ncRNAs).

In liquid cultures, strain TNT demonstrated rapid TNT transformation activity in minimal medium (113 mg liter^-1^ TNT, 0.1 mg liter^-1^ NH_4_Cl, and 5 g liter^-1^ glucose). Analyses of the draft genome confirmed the presence of genes coding for multiple TNT-transforming enzymes, including nitroreductase A, nitroreductase B, and *N*-ethylmaleimide (NEM) reductase. The NEM product is similar to NemA in *Escherichia coli* and to XenB and PetN of *Enterobacter cloacae*, and it is possibly responsible for nitrite release from TNT (5–9). Strain TNT uses nitrite as the sole N source under aerobic conditions, and genes for a complete nitrite assimilation pathway are present. This strain produces indole, is Voges-Proskauer positive, utilizes glycerol, glucose, fructose, and sucrose as carbon sources, and grows at 20°C. Genes for plant growth-promoting characteristics are present, corroborating results from phenotypic tests: auxin biosynthesis, acetoin production, 1-aminocyclopropane-1-carboxylate deaminase activity, siderophore production, phosphorous solubilization, and chemotaxis. *R. ornithinolytica* TNT is a promising strain for rhizoremediation applications.

### Nucleotide sequence accession numbers.

This whole-genome shotgun project has been deposited at DDBJ/EMBL/GenBank under the accession no. JHQH00000000. The version described in this paper is version JHQH01000000.

## References

[1] Shin SH Um Y Beak JH Kim S Lee S Oh MK Kim YR Lee J Yang KS 2013 Complete genome sequence of *Raoultella ornithinolytica* strain B6, a 2,3-butanediol-producing bacterium isolated from oil-contaminated soil. Genome Announc. 1(3):e00395-13. 10.1128/genomeA.00395-13 23814034 PMC3695430

[2] Chevreux B Wetter T Suhai S 1999 Genome sequence assembly using trace signals and additional sequence information, p 45–56 *In* Computer science and biology. Proceedings of the German Conf. on Bioinformatics, GCB ’99. GCB, Hannover, Germany

[3] Rissman AI Mau B Biehl BS Darling AE Glasner JD Perna NT 2009 Reordering contigs of draft genomes using the Mauve Aligner. Bioinformatics 25:2071–2073. 10.1093/bioinformatics/btp356 19515959 PMC2723005

[4] Angiuoli SV Gussman A Klimke W Cochrane G Field D Garrity G Kodira CD Kyrpides N Madupu R Markowitz V Tatusova T Thomson N White O 2008 Toward an online repository of Standard Operating Procedures (SOPs) for (meta)genomic annotation. Omics 12:137–141. 10.1089/omi.2008.0017 18416670 PMC3196215

[5] French CE Nicklin S Bruce NC 1998 Aerobic degradation of 2,4,6-trinitrotoluene by *Enterobacter cloacae* PB2 and by pentaerythritol tetranitrate reductase. Appl. Environ. Microbiol. 64:2864–2868 9687442 10.1128/aem.64.8.2864-2868.1998 PMC106784

[6] Williams RE Rathbone DA Scrutton NS Bruce NC 2004 Biotransformation of explosives by the old yellow enzyme family of flavoproteins. Appl. Environ. Microbiol. 70:3566–3574. 10.1128/AEM.70.6.3566-3574.2004 15184158 PMC427764

[7] Wittich RM Haïdour A Van Dillewijn P Ramos JL 2008 OYE flavoprotein reductases initiate the condensation of TNT-derived intermediates to secondary diarylamines and nitrite. Environ. Sci. Technol. 42:734–739. 10.1021/es071449w 18323095

[8] van Dillewijn P Wittich RM Caballero A Ramos JL 2008 Subfunctionality of hydride transferases of the old yellow enzyme family of flavoproteins of *Pseudomonas putida*. Appl. Environ. Microbiol. 74:6703–6708. 10.1128/AEM.00386-08 18791012 PMC2576699

[9] González-Pérez MM van Dillewijn P Wittich RM Ramos JL 2007 *Escherichia coli* has multiple enzymes that attack TNT and release nitrogen for growth. Environ. Microbiol. 9:1535–1540. 10.1111/j.1462-2920.2007.01272.x 17504490

